# The rise of the photosynthetic rate when light intensity increases is delayed in *ndh* gene-defective tobacco at high but not at low CO_2_ concentrations

**DOI:** 10.3389/fpls.2015.00034

**Published:** 2015-02-09

**Authors:** Mercedes Martín, Dolores M. Noarbe, Patricia H. Serrot, Bartolomé Sabater

**Affiliations:** ^1^Department of Life Sciences, University of Alcalá, Alcalá de HenaresSpain; ^2^Department of Physical Chemistry, University of Alcalá, Alcalá de HenaresSpain

**Keywords:** dispensable genes, fluctuating light, high CO_2_, *ndh* genes, photosynthesis

## Abstract

The 11 plastid *ndh* genes have hovered frequently on the edge of dispensability, being absent in the plastid DNA of many algae and certain higher plants. We have compared the photosynthetic activity of tobacco (*Nicotiana tabacum*, cv. Petit Havana) with five transgenic lines (*ΔndhF*, pr-*ΔndhF*, T181D, T181A, and *ndhF* FC) and found that photosynthetic performance is impaired in transgenic *ndhF*-defective tobacco plants at rapidly fluctuating light intensities and higher than ambient CO_2_ concentrations. In contrast to wild type and *ndhF* FC, which reach the maximum photosynthetic rate in less than 1 min when light intensity suddenly increases, *ndh* defective plants (*ΔndhF* and T181A) show up to a 5 min delay in reaching the maximum photosynthetic rate at CO_2_ concentrations higher than the ambient 360 ppm. Net photosynthesis was determined at different CO_2_ concentrations when sequences of 130, 870, 61, 870, and 130 μmol m^-2^ s^-1^ PAR sudden light changes were applied to leaves and photosynthetic efficiency and entropy production (S_g_) were determined as indicators of photosynthesis performance. The two *ndh*-defective plants, *ΔndhF* and T181A, had lower photosynthetic efficiency and higher S_g_ than *wt*, *ndhF* FC and T181D tobacco plants, containing full functional *ndh* genes, at CO_2_ concentrations above 400 ppm. We propose that the Ndh complex improves cyclic electron transport by adjusting the redox level of transporters during the low light intensity stage. In *ndhF*-defective strains, the supply of electrons through the Ndh complex fails, transporters remain over-oxidized (specially at high CO_2_ concentrations) and the rate of cyclic electron transport is low, impairing the ATP level required to rapidly reach high CO_2_ fixation rates in the following high light phase. Hence, *ndh* genes could be dispensable at low but not at high atmospheric concentrations of CO_2_.

## INTRODUCTION

Some 30 years after their discovery (Ohyama et al., 1986; Shinozaki et al., 1986), the functional role of the 11 plastid-encoded *ndh* genes (which are homologous to genes encoding components of Complex I of the mitochondrial respiratory chain) is still a mystery. Among eukaryotic algae, only a few Prasinophyceae and all Charophyceae (the green algae related to higher plants) contain *ndh* genes (Martín and Sabater, 2010). Most photosynthetic land plants contain the *ndh* genes, which are absent in parasitic non-photosynthetic species of the genera *Cuscuta*, *Epiphagus*, *Orobanche* and the Orchidaceae family. This suggests that the thylakoid Ndh complex, encoded by the 11 plastid *ndh* genes and an as yet unknown number of nuclear genes, has a role in land plant photosynthesis. However, plastid DNAs of the photosynthetic Gymnosperms Pinaceae and Gnetales and of a few species scattered among angiosperm genera, families, and orders (e.g., *Erodium*, Ericaceae, Alismatales,…) lack *ndh* genes (Braukmann et al., 2009; Blazier et al., 2011; Braukmann and Stefanović, 2012), which suggests that *ndh* genes could be dispensable under certain environments. The 11 *ndh* genes account for about 50% of all C to U editing sites identified in the transcripts of plastid genes (Tillich et al., 2005), which again suggests that dispensable *ndh* genes in the ancestors of extant plants accumulated inactivating mutations (Martín and Sabater, 2010). Of these, T to C mutations have been neutralized by C to U transcript editing in most extant plants which presumably recuperates the functionality of the *ndh* genes. Thus, tottering on the edge of dispensability, the *ndh* genes provide an excellent opportunity to test the natural selection of photosynthesis-related genes during plant evolution.

It is widely accepted that the Ndh complex is located in the stromal thylakoids (Casano et al., 2000, 2004; Lennon et al., 2003), transfers electrons to plastoquinone and is involved in cyclic electron transport. However, two different electron donors have been proposed: reduced ferredoxin and NADH. Ferredoxin as the electron donor would imply a role of the Ndh complex providing a cyclic photosynthetic electron transport (Yamamoto et al., 2011) in addition to the commonly accepted model in which ferredoxin directly donates electrons to the PQ/*cyt.b_6_f* intermediary electron pool (Kurisu et al., 2003). However, as pointed out by Nandha et al. (2007) in a similar assay with the PGR5 protein, the rate of plastoquinone reduction is too low in the assay of the Ndh complex with reduced ferredoxin as electron donor. In contrast, spectrophotometric assays of activity and zymogram of NADH dehydrogenases after native electrophoresis, combined with immunodetection with antibodies raised against protein encoded by chloroplast *ndh* genes indicate NADH as the electron donor in different plants (Cuello et al., 1995; Corneille et al., 1998; Sazanov et al., 1998; Casano et al., 2000; Díaz et al., 2007; Martín et al., 2009; Serrot et al., 2012). Accordingly, the Ndh complex, in concerted action with electron draining reactions (Mehler, superoxide dismutase/peroxidase and terminal oxidase) in chlororespiration, would contribute to adjust (poise) the redox level of the cyclic photosynthetic electron transporters (Casano et al., 2000; Joët et al., 2002; Rumeau et al., 2007), hence optimizing (Heber and Walker, 1992) the transport rate necessary for cyclic photophosphorylation and, in general, the thylakoid polarization and lumen acidification that is also required to avoid the damages caused by excess light by dissipating energy through zeaxanthin (Eskling et al., 2001; Karpinski et al., 2001; Minagawa, 2013) in the process of non-photochemical quenching. Accordingly, by poising the redox level of the cyclic electron transporters, the Ndh complex contributes to the protection against photo-oxidative-related stresses (Martín et al., 2004; Sabater and Martín, 2013). The activity superoxide dismutase, which is key for electron draining, decreases in adult-senescent photosynthetic tissues, when the over-expression of the *ndh* genes results in an over-reduction of electron transporters which triggers the accumulation of reactive oxygen species inducing cell death (Zapata et al., 2005; Nashilevitz et al., 2010; Nilo et al., 2010; Sabater and Martín, 2013).

Related to the function of the Ndh complex in cyclic electron transport, questions remain on the extent the *ndh* genes improve (if such is the case) photosynthesis and on the environmental conditions that made *ndh* genes dispensable in certain plant lines. Apart from a certain weakening under different stress conditions, transgenic plants defective in *ndh* genes usually show normal growth (Burrows et al., 1998; Martín et al., 2004). However, the information provided by transgenic *ndh*-defective plants is sometimes debatable. Only a few of the claimed nuclear *ndh* genes have been unambiguously demonstrated to encode Ndh components (Darie et al., 2005; Rumeau et al., 2005; Shimizu et al., 2008). In fact, the basic respiratory complex I found in archaeal and eubacterial kingdoms may be functional with only 11 subunits (Moparthi et al., 2014) homologous to those encoded by the 11 plastid *ndh* genes. Frequently, *Arabidopsis* nuclear mutants defective in the thylakoid Ndh complex are affected in subunit assembly, plastid *ndh* transcript processing and, in general, processes that can have pleiotropic effects on several chloroplast functions (Meurer et al., 1996). On the other hand, the obtainment of homoplastomic plastid *ndh* transgenics is highly improbable. Although efficient technologies are available that insert foreign sequences, the large background provided by 100s copies of plastid DNA (Rauwolf et al., 2010; Matsushima et al., 2011) in mesophyll cells makes the selection of homoplastomic transformed cells difficult, even after several culture cycles. DNA-blot hybridizations are not sufficiently sensitive to establish homoplastomy, and even more sensitive approaches such as PCR amplification could be insufficient. Rapid replication of non-transformed plastid DNA makes it difficult to maintain plants with a high proportion of plastid *ndh* defective DNA for several generations, unless selective culture conditions are regularly maintained during the 2 or 3 weeks after germination. Although not homoplastomic, the low level of non-transformed DNA has allowed us to investigate the functional properties of transformed tobacco plants that contain a high proportion of plastid DNA with defective *ndh* genes and show low amounts or malfunctioning thylakoid Ndh complex (Martín et al., 2004, 2009; Zapata et al., 2005).

The functioning of the photosynthetic machinery under rapidly changing environmental conditions (mainly light intensity) is recently receiving considerable research interest (Martín et al., 2009; Tikkanen et al., 2012; Garab, 2014). Photosystem I protection, cyclic electron transport and the control of reactive oxygen species require strategies that are being intensely investigated and are different from those under constant high light (Suorsa et al., 2013). However, little is known on the final effect of rapidly fluctuating light on net photosynthesis. The slight delay in reaching full photosynthetic rates in transgenic *ndh*-defective tobacco plants after sudden increases of light intensity (Martín et al., 2009) prompted us to investigate the contribution of the *ndh* genes to suppress that delay and the consequences on the photosynthetic efficiency and S_g_, as measures of fitness (Sabater, 2006; Marín et al., 2014), at different CO_2_ concentrations and rapidly fluctuating light intensity. To maintain the low entropy associated to leaf organization (Ksenzhek and Volkov, 1998; Davies et al., 2013; Marín et al., 2014) the entropy produced in photosynthesis must be exported, which increases the global entropy as required for all irreversible processes (Schrödinger, 1944). Therefore, comparisons of the entropy produced in photosynthesis by *wt* and *ndh*-deficient plants with the negative entropy associated to cell organization would help to evaluate the advantages provided by the *ndh* genes.

Measurements of net photosynthetic rates revealed that the increase of the rate of photosynthesis when the intensity of light suddenly increases is delayed in *ndh*-defective plants when compared with *wt* and control transformed (*ndhF* FC) plants at high but not at low concentrations of CO_2_. Probably, by balancing the redox level of transporters, the Ndh complex maintains high rates of photosynthetic cyclic electron transport during the low light intensity stage to maintain thylakoid polarization and the ATP level required to protect photosynthetic machinery and the rapid response of photosynthetic rate when light suddenly increases in the following stage. The consequence is comparatively low photosynthetic efficiency and high S_g_ in *ndh*-deficient tobacco plants under rapid fluctuating light and high concentrations of CO_2_, which suggests that the *ndh* genes could be dispensable at low atmospheric concentrations of CO_2_, but not at higher CO_2_ concentrations.

## MATERIALS AND METHODS

### PLANTS CULTURE

Most assays were performed with *wt* tobacco (*Nicotiana tabacum*, cv. Petit Havana) and transgenics defective in the *ndhF* gene by intragenic insertion of the spectinomycin-selection gene *aadA* (Koop et al., 1996; Martín et al., 2004, 2009; *ΔndhF* and pr-*ΔndhF* described later). Additional assays were carried out with different tobacco plants where the *aadA* selection gene was inserted upstream of the *ndhF* gene (Martín et al., 2009) maintaining intact *ndhF* gene (*ndhF* FC, control) or site-directed mutated: T181A and T181D, in which the ^541^ACT^543^ triplet encoding the phosphorylatable Thr-181 of the NDH-F subunit has been substituted by GCT and GAT encoding alanine and aspartic acid, respectively.

Tobacco plants were cultured as described Martín et al. (2009). Seeds from non-transformed *wt* tobacco were sown in pots with compost soil substrate, germinated and grown in a glasshouse. Seeds from transformed plants were aseptically germinated and grown for 1–2 months in sterile Murashige/Skoog (MS) agar-solidified medium supplemented with 600 mg L^-1^ spectinomycin. Plantlets were transplanted to compost soil substrate in pots under controlled glasshouse conditions and irrigated with MS. The genetic identity of the different tobacco plants was regularly tested by primer-directed amplification of appropriate plastid DNA regions, size determination, and sequencing (Martín et al., 2004, 2009). Since 2002 (for *ΔndhF*) and 2007 (for the other transgenics), new seed generations of each transformed tobacco plant were produced at least once a year by completing the life cycle of the original transformed plants (Martín et al., 2004, 2009), obtained as detailed in Supplementary Materials. Sample seeds of *ΔndhF*, T181D, and *ndhF* FC are available from authors upon request.

### MEASUREMENT OF NET PHOTOSYNTHESIS, TRANSPIRATION RATES, AND CHLOROPHYLL FLUORESCENCE INDUCTION

Photosynthesis and transpiration rates were determined in the glasshouse at 25°C in 6.25 cm^2^ regions of intact fully expanded healthy leaves (containing ∼20 μg chlorophyll cm^-2^), of the mid-stem of plants at the beginning of flowering. Leaf was, fitted on the chamber of the LCpro+ portable photosynthesis system (ADC BioScientific Ltd., Hertfordshire, UK) as previously described Martín et al. (2009) and Marín et al. (2014), except for the CO_2_ concentration that, having been programmed as fixed during the light sequence treatment was varied. Net photosynthetic activity (in μmol consumed of CO_2_ m^-2^ s^-1^) and transpiration rate (in mmol of H_2_O m^-2^ s^-1^) were measured during the light sequence treatment where the intensity (in μmol m^-2^ s^-1^ PAR, at leaf surface) abruptly changed according to the sequence: 15 min acclimation at 130, 6 min at 870, 6 min at 61, 6 min at 870, and 6 min at 130 μmol m^-2^ s^-1^ PAR. Data collected each min and at light intensity transitions were directly represented using the GraFit Erithacus software (Surrey, UK) and the Origin software (Princeton, NJ, USA). Registered data indicated that the sub-stomatal CO_2_ concentration was stabilized (<5% variation) from the end of 15 min acclimation through the following 24 min incubation. Experiments were repeated 2–10 times.

The rates of net photosynthesis and transpiration determined in attached leaf sections varied during different days, probably due the variable environmental factors affecting the whole plant. However, the relative rates with different CO_2_ concentrations, determined in the same day, did not differ by more than 5% after 2–10 determinations during the year in *wt* and the control *ndhF* FC tobacco lines. Therefore, absolute rates showed in each figure correspond of measurements in the same day, when 3–5 CO_2_ concentrations were assayed. Figures are representative of 2–10 experiments. The influence of the concentration of CO_2_ on the photosynthetic efficiency and the production of entropy was expressed relative to the values at the reference 360 ppm CO_2_ and all experimental results were represented. More details on the statistical significance of the results are discussed in appropriate sections.

Fluorescence assays were carried out in the glasshouse with similar leaves as those used in photosynthesis rate determinations. Chlorophyll fluorescence changes were measured with an Opti-Sciences (ADC BioScientific Ltd., Hertfordshire, UK) OS1-FL modulated chlorophyll fluorometer. Standard assay was used with relative minimum and high light intensities optimized to show differences among *ndh* mutants (Martín et al., 2009). Leaf disk regions were dark-adapted with clips for 30 min after which they received 2 min minimum light (0.1 μmol m^-2^ s^-1^ PAR) followed by 5 min higher relative light (0.15 μmol m^-2^ s^-1^ PAR) and 9 min again of minimum light. 0.8 s saturating flashes (5,000 μmol m^-2^ s^-1^ PAR) were applied at 1, 3, 4, 5, and 6 min of light incubation. Fluorescence was recorded each 0.1 s and collected data were represented using the GraFit Erithacus software (Surrey, UK) and the Origin software (Princeton, NJ, USA). Assays repeated at least three times showed no significant differences. Yield of quantum efficiency (Y), of light energy absorbed by photosystem II which is used in photosynthetic electron transport, was calculated as Y=(Fms–Fs)/Fms. Where: Fms is maximal fluorescence and Fs is variable fluorescence under steady state.

### PHOTOSYNTHETIC EFFICIENCY AND ENTROPY PRODUCTION

As detailed previously (Marín et al., 2014), photosynthetic efficiency (η, the fraction of absorbed radiant energy converted to biomass chemical energy) is: η = 100 E_BI_/E_in_, where E_BI_ is the chemical Gibbs energy of the net photosynthesis products stored as biomass (CH_2_O) and E_in_ the absorbed PAR measured at leaf surface and corrected by the 7% transmitted. The entropy generated (S_g_) was calculated from net CO_2_ fixation data, dimensions of the experimental design, Gibbs free energy and entropy values in data banks and conventional thermodynamics. The entropy generation was expressed per J (Joule) of biomass chemical energy generated: S_g_/E_BI_.

For each photosynthesis assay, the integrated net CO_2_ consumed over the last 24 min of light incubation and the intermediate 12 min light phases (at 61 and the following at 870 μmol m^-2^ s^-1^ PAR) were converted to C-equivalent biomass (CH_2_O) according to the reaction:

$${CO}_{2}(gas)+H_{2}O(liquid)\to 1/6C_{6}H_{12}O_{6}(solid)+O_{2}\left(gas\right)$$

The integrated fixed CO_2_ (mol m^-2^) was multiplied by the equivalence:

$$\begin{array}{c} E_{BI}^{\prime }=\Delta G=\Delta G^{0}+RT\,\;\ln\left(P_{02}/P_{CO2}\right)\\ \;\;\;\;\;\;=475.9-RT\,\;\ln(10^{-6}\times \left[{CO}_{2}\right])kJ{mol}^{-1} \end{array}$$

(calculated with: ΔG^0^ = 479.8 kJ mol^-1^, R = 8.314 J mol^-1^ K^-1^, T = 298 K, P_O2_ = 0.21 bar and where [CO_2_], in the later equation, is in ppm) to obtain the energy (E_BI_) stored as biomass and synthesized per square meter.

Similarly, the associated production of entropy (S′_BI_) was determined by:

$$\begin{array}{c} S_{BI}^{\prime }=\Delta S=\Delta S^{0}-R\,\ln\left(P_{02}/P_{CO2}\right)\\ \;\;\;\;\;\;\;\;=-30.2+R\,\ln(10^{-6}\left[{CO}_{2}\right])JK^{-1}{mol}^{-1} \end{array}$$

By considering a PAR λ mean of 550 nm and applying the equivalence: radiant energy (J) = 119.3 × 10^6^/λ(nm), the absorbed PAR energy (E_in_) was estimated 140.2 kJ m^-2^ and 67.6 kJ m^-2^ for the last 24 min and the intermediate 12 min light phases, respectively. Their associated entropies were determined as that of non-diffuse sunlight by considering an effective temperature of 5000 K for solar radiation (Ksenzhek and Volkov, 1998) by:

$$\begin{array}{c} S_{in}=E_{in}/5000\,\;JK^{-1}.28.0\,\;\;and\,\;\;13.5\,\;\;JK^{-1}m^{-2}\\ \;\;\;\;\;\;\;\;\;\;for,respectively\,\;\;24\,\;\;and\,\;\;12\,\;\;\min\;light\,\;\;\;phase. \end{array}$$

The S_g_ was obtained by subtracting S_in_ from the sum of all forms of energy wasted which total E_in_ – E_BI_ divided by the absolute temperature, T, plus the entropy of the biomass produced (S_BI_), thus:

$$S_{g}=(E_{in}-E_{BI})/T+S_{BI}-E_{in}/5000\,\;JK^{-1}m^{-2}$$

Measurements and derived calculations at different CO_2_ concentrations were referred as percentages of the results at 360 ppm CO_2_ obtained with the same plant and group of experiments.

### OTHER DETERMINATIONS AND ASSAYS

DNA isolation, PCR amplification and agarose gel electrophoresis were performed as described previously (Martín et al., 2004). Zymograms and immunoassays related to the thylakoid Ndh complex were also performed as described previously (Martín et al., 2009).

## RESULTS

### TOBACCO LINES WITH PARTIAL RECOVERY OF *ndhF* GENE COPIES

**FIGURE 1:**
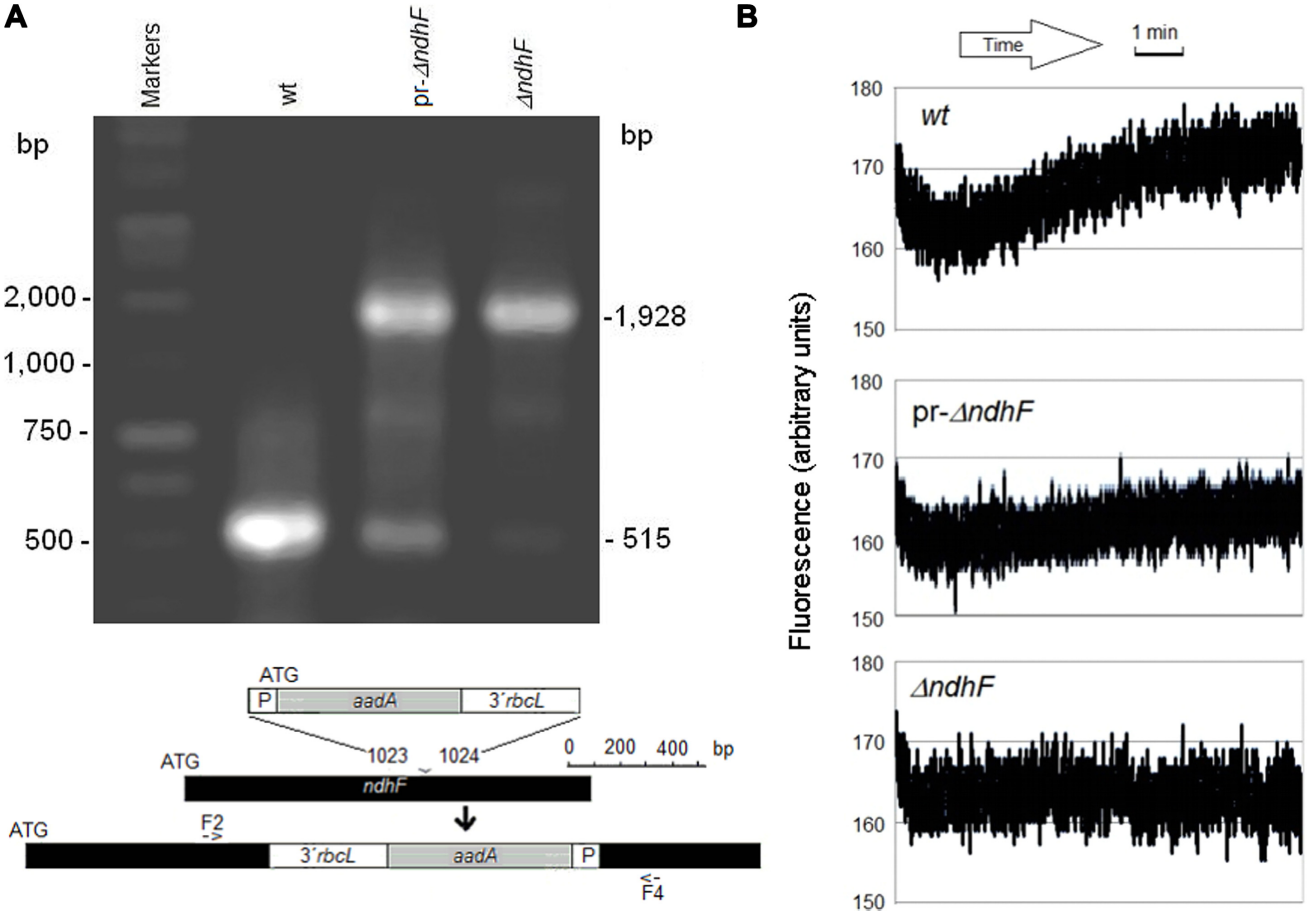
**Genetic identification and fluorescence properties of pr-*ΔndhF* tobacco. (A)** PCR amplification products of plastid DNAs of *wt*, pr-*ΔndhF,* and *ΔndhF* tobacco plants using primers F2/F4 (Serrot et al., 2012) for the *ndhF* gene sequence (bottom map). Sizes of the main amplified fragments and of some markers are indicated on the left and right, respectively. **(B)** Chlorophyll fluorescence traces of *wt*, pr-*ΔndhF,* and *ΔndhF* tobacco plants after relative high to minimum light transition. Assays were performed with intact tobacco leaves as described Martín et al. (2009). The traces shown are of fluorescence readings every 0.1 s during the final 9 min of minimum light. Vertical axes show the relative fluorescence readings.

In addition to previously described *wt*, *ndhF* FC, *ΔndhF*, T181A, and T181D tobacco plants (Martín et al., 2009), we assayed partially reverted phenotypes of *ΔndhF* (pr-*ΔndhF*) that we have found among descendants of the *ndh*-deficient *ΔndhF* tobacco transgenic, as identified by the increase of the 515 bp PCR-amplified band (**Figure 1A**, lane pr-*ΔndhF*) characteristic (Martín et al., 2004; Zapata et al., 2005) of the non-transformed plastid DNA of *wt* (**Figure 1A**, lane *wt*). The relative intensities of the amplified 1,928 and 515 bp bands should approximate and respectively mirror the relative abundance of transformed (*ΔndhF*) and non-transformed (*wt*) plastid DNA molecules among the 100s of DNA copies contained in a single mesophyll cell. The presumably low proportion of the functional *ndhF* gene in pr-*ΔndhF* only slightly permitted the recovery of the clear post-illumination fluorescence increase characteristic of *wt* (**Figure 1B**) that is absent in *ndh* deficient plants (Burrows et al., 1998; Martín et al., 2004). Accordingly, pr-*ΔndhF* phenotypes showed a thylakoid Ndh-dependent NADH dehydrogenase activity which was lower than in *wt* but higher than in *ΔndhF* transgenic (not shown). In contrast to the clearly delayed leaf senescence phenotype of *ΔndhF* (Zapata et al., 2005), pr-*ΔndhF* showed only slight delayed leaf senescence in comparison to *wt* tobacco (Figure S1). The frequency of pr-*ΔndhF* phenotype is increasing in successive offspring derived from the original *ΔndhF* tobacco (despite the presence of spectinomycin during the initial culture of *ΔndhF*). Conceivably, unknown factors favor the replication of the few remaining copies of *wt* plastid DNA in *ΔndhF* tobacco over the transformed molecules defective in the *ndhF* gene. Although we are not yet able to control its emergence or its inheritance, the finding of pr-*ΔndhF* phenotype provides an additional retro-mutant control that confirms the involvement of *ndh* genes in photosynthesis and other processes. In the future, the ability to control (and determine by quantitative PCR) the *wt* to *ΔndhF* plastid DNA ratio will provide a deeper understanding of the influence of the copy proportion of the plastid *ndh* gene in different processes.

### PHOTOSYNTHETIC RATES UNDER FLUCTUATING LIGHT INTENSITIES. EFFECT OF THE CONCENTRATION OF CO_2_

**FIGURE 2:**
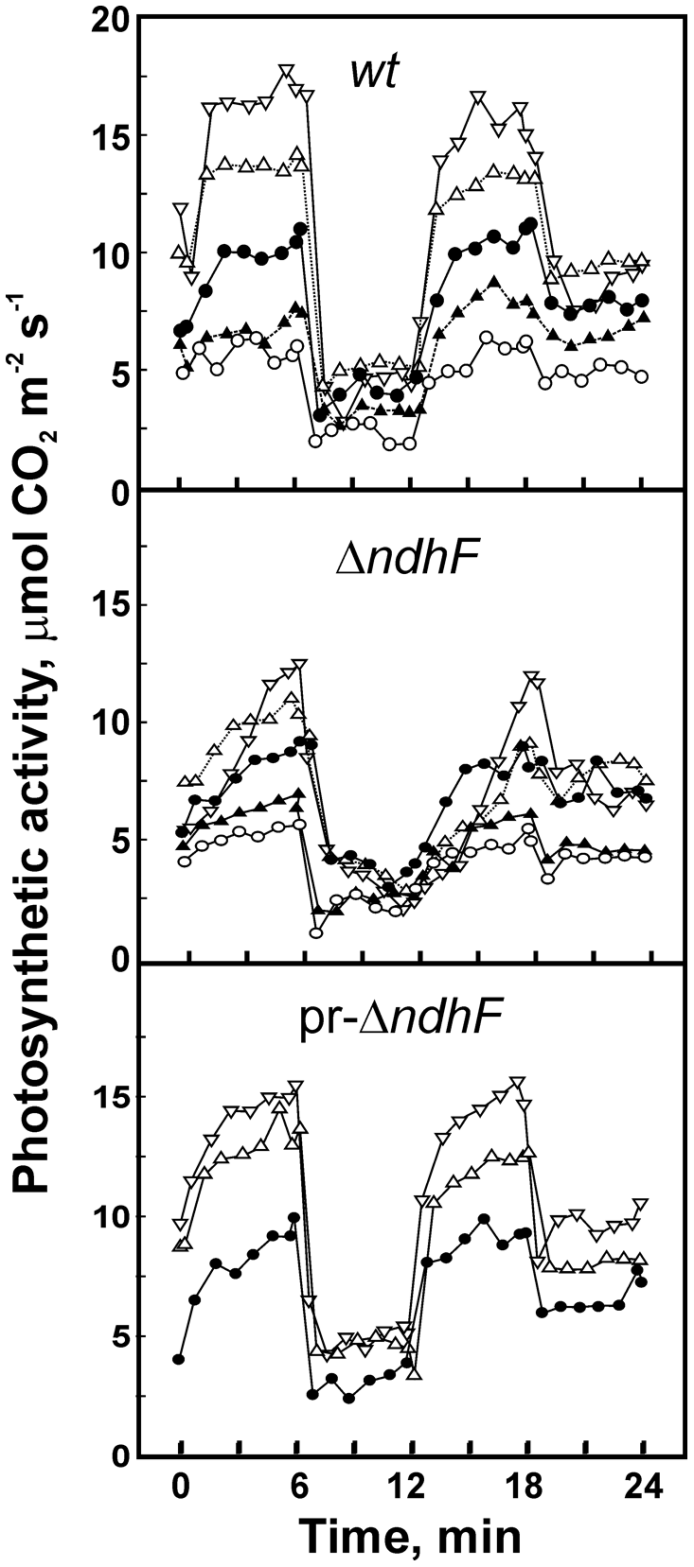
**Effect of CO_**2**_ concentration on photosynthetic rate under fluctuating light.** Assays were carried out with *wt*, *ΔndhF,* and pr*-ΔndhF* tobacco leaves and the figure shows representative graphics of 3–8 different series of experiments. After a 15 min acclimation at 130 μmol m^-2^ s^-1^ PAR, the leaf region entrapped in the climatic chamber was subjected to successive 6 min periods (starting at 0 time in the figure) of light intensities, abruptly changing according to the sequence 870, 61, 870, and 130 μmol m^-2^ s^-1^ PAR. The concentration of CO_2_ varied for each sequence of light treatments as adjusted to (in ppm): 260 (-◯-), 312 (-▲-), 365 (-●-), 475 (-Δ-), and 625 (-▽-).

In the field, leaves are exposed to frequent and rapid changes in light intensity (0 to about 2,500 μmol m^-2^ s^-1^ PAR) due to transitory shadow produced by clouds, by other leaves fluttering in the wind and by wandering animals (Külheim et al., 2002). To investigate the effect of rapid light intensity variations on the photosynthetic performance of leaves, we established a reference light fluctuation incubation consisting of 15 min of leaf acclimation at 130, followed by four 6-min light phases of 870, 61, 870, and 130 μmol m^-2^ s^-1^ PAR at leaf surface. Rates of net photosynthesis varied for the same plant from 1 day and leaf to another. However, relative photosynthetic rates for different CO_2_ concentrations were highly reproducible for a same tobacco line with differences not higher than 5% and result in similar shape of the rate-time curves characteristic for each CO_2_ concentration. Therefore, we determined photosynthetic rate curves for up to five different CO_2_ concentration assays carried out successively with the same leaf section by changing the setting of the CO_2_ concentration. The 15 min acclimation was repeated for each CO_2_ concentration and the order of the assays with different CO_2_ concentrations (increasing or decreasing) did not affect the responses of photosynthesis rates. Experiments were repeated without significant differences 2–10 times and each graph in the following **Figure 2** corresponds to one representative group of assays carried out in the same day with each plant and variable concentrations of CO_2_.

**Figure 2** shows photosynthetic rates during the four light phases after the 15 min acclimation of *wt*, one pr-*ΔndhF* phenotype and *ΔndhF* tobacco plants at different CO_2_ concentrations. In most repeated experiments (Figure S2), *wt* showed the highest and *ΔndhF* the lowest photosynthetic rates but, as stated above, no definitive conclusion could be drawn from differences of activity among the three plants. However, differences in the time with which each plant reaches maximum activity after light intensity increased to 870 μmol m^-2^ s^-1^ were highly reproducible. In contrast to *wt*, which rapidly reached the maximum photosynthetic rate when light increased from 130 or 61 to 870 μmol m^-2^ s^-1^ (**Figure 2**, upper box), *ΔndhF* (middle box) showed a considerable delay in reaching the maximum photosynthetic rate at CO_2_ concentrations higher than the ambient 360 ppm and, paradoxically, the photosynthetic rate of this transgenic was higher at 475 (-Δ-) than at 615 (-▽-) ppm CO_2_ during the first 3 min after the transition from acclimation 130 to 870 μmol m^-2^ s^-1^. Furthermore, during the first 3–5 min after the transition from 61 to 870 μmol m^-2^ s^-1^ (12 to 18 min in **Figure 2**) the photosynthetic rate was higher at 365 (-●-) than at 475 (-Δ-) and 615 (-▽-) ppm CO_2_. By comparison, during the fluctuating light incubation, the rate of photosynthesis in *wt* was always higher the higher the concentration of CO_2_. At low CO_2_ concentrations, 260 to 312 ppm (– and –, respectively), there were no detectable differences between *wt* and *ΔndhF* tobacco plants in the increase of the rate of photosynthesis after transition to high light.

Figure S2 shows photosynthetic rate versus time curves for several groups of assays (each group corresponds to experimental determinations carried out within the same day and leaf) with *wt* (four groups), pr-*ΔndhF* (two groups), and *ΔndhF* (three groups) tobacco plants. Figure S2 shows the phenotypic variability of the various *ΔndhF* strains. Rate curves are highly reproducible within the same day and leaf (see groups *wt*-3, *wt*-4, and pr-*ΔndhF*-2 in Figure S2) for the same CO_2_ concentration, although absolute rate values for the same plant and similar CO_2_ concentration significantly vary among the different days of assay (compare in Figure S2 the high activity at 470 ppm CO_2_ in *wt*-1 with the low activity in *wt*-4 group of assays).

The rates of photosynthesis in pr-*ΔndhF* were determined at CO_2_ concentrations of 365 ppm and higher (**Figure 2**, bottom box) and the curves were closer to those of *wt* or to *ΔndhF*, probably dependant on the relative *ndhF* gene dose with respect to *ΔndhF* tobacco. Therefore, these results support that the products of intact functional *ndh* genes improve the photosynthetic performance at fluctuating light intensities at higher than ambient CO_2_ concentrations. The slow increase of the rate of photosynthesis at high concentrations of CO_2_ in *ΔndhF* is not due to an indirect effect of plastid DNA transformation because the control *ndhF* FC transgenic tobacco, containing the inserted *aadA* gene and intact *ndhF* gene, did not show delay in reaching high photosynthetic rate when assayed up to 535 ppm CO_2_ (Figure S3).

**FIGURE 3:**
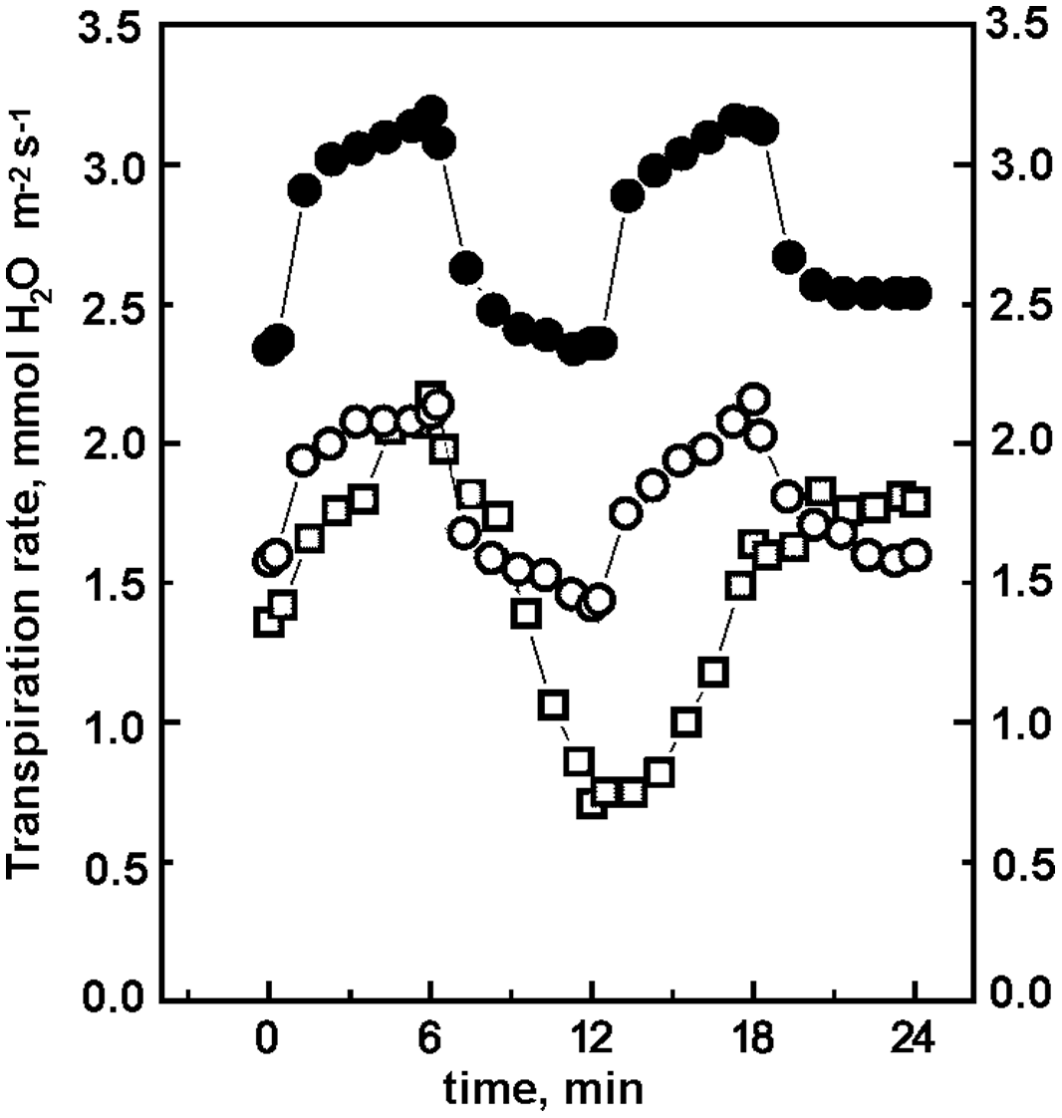
**Transpiration rates of *wt*, *ΔndhF,* and pr-*ΔndhF* tobacco plants at 470 ppm CO_**2**_ and fluctuating light.** After 15 min acclimation at 130 μmol photon m^-2^ s^-1^ PAR, the leaf region entrapped in the climatic chamber was subjected to successive 6 min periods (starting at 0 time in the figure) of light intensities, abruptly changing according to the sequence 870, 61, 870, and 130 μmol photon m^-2^ s^-1^ PAR. Transpiration rates were measured as described in methods. *wt* (-●-), *ΔndhF* (-□-), and pr-*ΔndhF* (◯).

The slow increase of the rate of photosynthesis at high concentrations of CO_2_ cannot be attributed to an impaired stomatal response of *ΔndhF* with respect to *wt*. We found (Marín et al., 2014) that in *wt* tobacco, under the successive 6 min periods at 870, 61, 870, and 130 μmol m^-2^ s^-1^ PAR and different concentrations of CO_2_, the rate of photosynthesis varied strongly and rapidly between a minimum and an approximately 5-fold higher maximum; a result similar to that shown in **Figure 2**. In contrast, the rates of transpiration and the stomatal conductance changed slowly with a maximum that barely doubled minimum values. In this work, we found similar results for all tobacco plants: the rates of transpiration and the stomatal conductance change slower than the rates of photosynthesis and the span between maximum and minimum values is significantly narrower for transpiration and conductance than for photosynthesis. As an example, **Figure 3** shows that at 470 ppm CO_2_ the changes in transpiration (increase at high light and decrease at low light intensities) were slower in *ΔndhF* than in *wt* tobacco. Transpiration rates mirrored stomatal conductance changes determined in parallel assays (not shown). Similarly to photosynthesis, the transpiration responses in partially reverted pr-*ΔndhF* tobacco lay between those of *wt* and *ΔndhF* plants. The comparison with **Figure 2** indicates that, in both *wt* and *ΔndhF*, rate responses are slower for transpiration than for photosynthesis. In general, there is an inverse relation between the internal CO_2_ concentration in the leaf and the stomatal opening and, consequently, transpiration (Wheeler et al., 1999). As the internal concentration of CO_2_ is lower at higher photosynthetic activity, it seems likely that the slower photosynthetic response in *ΔndhF* than in *wt* is responsible for the even slower transpiration response of *ΔndhF* than of *wt*, and not the opposite.

### DETERMINATIONS OF PHOTOSYNTHETIC EFFICIENCY AND ENTROPY PRODUCTION

We evaluated the efficiency of the conversion of radiant energy to chemical energy (biomass) and the S_g_ of *wt* and mutant plants both during the total 24 min and for the intermediate 12 min light treatments (6 min at 61 and 6 min at 870 μmol m^-2^ s^-1^ PAR). Obviously, the relative differences between *wt* and *ΔndhF* were higher for the intermediate 12 min than for the 24 min incubation.

*wt*, *ΔndhF*, pr-*ΔndhF*, point mutant transgenics T181A and T181D and control *ndhF* FC (containing the *aadA* spectinomycin resistance gene near the 5′end of the unmodified *ndhF* gene) plants were assayed. In T181A and T181D, the phosphorylatable threonine-181 is substituted by alanine and aspartic acid, respectively. Thus, the thylakoid Ndh complex in T181A cannot be activated by phosphorylation, whereas the negative charge of aspartic acid (D) in T181D mimics the activation effect of the threonine phosphorylation in *wt*, resulting in a highly active Ndh complex (Martín et al., 2009). As Figure S2 (for *wt*, pr-*ΔndhF,* and *ΔndhF*), S3 (for *ndhF* FC), and S4 (for T181D and T181A) show rate curves obtained from several groups of assays with T181D and T181A tobacco plants. Interestingly, T181D shows slight delay to reach full photosynthesis rate at low CO_2_ concentration (T181D-1 in Figure S4), but similarly to *wt* and in contrast to *ΔndhF* (**Figure 2**) and T181A (T181A-1, T181A-3, and T181A-4 in Figure S4) no delay at high CO_2_. Therefore, in agreement with previous enzyme determinations (Martín et al., 2009), the Ndh complex of T181D is probably always active due to the negative charge of aspartate (D), while the Ndh of *wt* tobacco requires 181-threonine phosphorylation. Conceivably, de-regulated hyperactive Ndh complex in T181D tobacco could over-reduce cyclic electron transporters at low CO_2_ concentrations, when the electron draining by Benson–Calvin cycle is low. The consequences would be low rate of cyclic transport and over production of reactive oxygen species. In this aspect, T181D tobacco would provide an interesting tool for further investigation of the redox and protein phosphorylation control of photosynthetic electron transport. The higher photosynthetic performance of T181D than of non-phosphorylatable T181A tobacco at high CO_2_ concentration is also appreciated when comparing efficiency and S_g_ at different CO_2_ concentrations.

Since the experimental approach did not provide a reliable comparison of absolute photosynthetic measurements on different days, a reference assay at 360 ppm of CO_2_ (sometimes interpolated from data at very similar concentrations) was always carried out within each group of experiments. Therefore, integrated net CO_2_ fixations, over the last 24 min or the intermediate 12 min of light incubation time, with the other 2–4 CO_2_ concentrations (assayed in the same group of experiments) were referred to the CO_2_ fixation at 360 ppm CO_2_ and photosynthetic efficiency and S_g_ were expressed as percentages of the respective values at 360 ppm CO_2_. This approach allowed us to combine results from about 35 groups of assays carried out different days with the six tobacco lines totalizing 139 rate-time curves.

**FIGURE 4:**
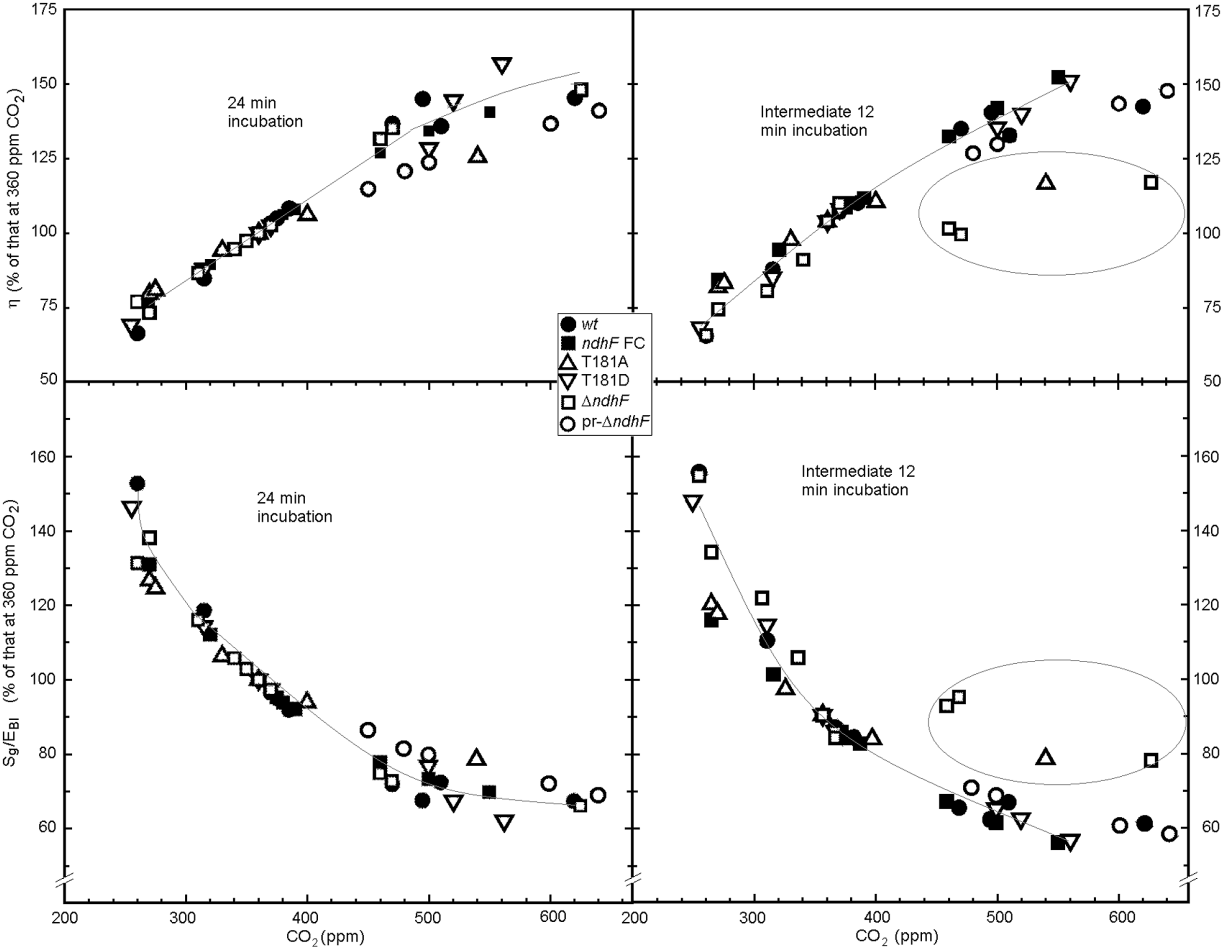
**Effect of the concentration of CO_**2**_ on photosynthetic efficiency (η) and S_**g**_/E_**BI**_ of different tobacco plants.** As explained in the thermodynamic background, photosynthetic efficiency is the ratio of chemical energy (E_BI_) stored as photosynthesized biomass to the PAR energy absorbed by the leaf. S_g_/E_BI_ is the ratio of the total entropy produced to the chemical energy (E_BI_) stored as photosynthesized biomass. Calculations were performed for the total 24 min (left boxes) and the 12 min intermediate (right boxes) light incubation treatments. The represented values of η and S_g_/E_BI_ at different CO_2_ concentrations are the percentages with respect to corresponding values at 360 ppm CO_2_ (references at 360 ppm are in the range of 3.6% η and 0.08 K^-1^ S_g_/E_BI_ for all plants). The concentrations of CO_2_ represented are those determined by the LCpro+ photosynthesis system in the leaf chamber and could slightly change with respect to those programmed. As repeated determinations for very similar CO_2_ concentrations in a same tobacco produced very close points, only the mean value is represented. Therefore, points in the figure result from 139 different assays and several are the mean of 2–4 independent determinations. Inserted gray lines were obtained by second degree polynomial fitting of the experimental points corresponding to *wt*, *ndhF* FC, T181D tobacco plants that contain full dose of functional *ndhF* gene. Encircled points in left boxes correspond to those of *ΔndhF* and T181A at high CO_2_ concentrations.

Photosynthetic efficiency η increased almost linearly with the concentration of CO_2_ (upper boxes of **Figure 4**) up to 400 ppm in an impressively very similar manner in all plants, which supports the statistical relevance of the approach. For CO_2_ concentrations higher than 400 ppm significant h differences were observed among tobacco lines: in most assays pr-*ΔndhF* (◯) and T181A (Δ) showed lower η (and consequently higher S_g_/E_BI_) during the 24 min incubation period (left boxes; compare with the gray line corresponding to plants with full functional *ndhF* gene described in the legend of **Figure 4**). For the intermediate 12 min incubation period (right boxes) when a strong rise from 61 to 870 μmol m^-2^ s^-1^ PAR took place, all assays with *ΔndhF* (□, three assays) and T181A (Δ, one assay; encircled) failed to significantly increase η at concentrations of CO_2_ higher than 400 ppm. For the intermediate 12 min period at higher than 400 ppm CO_2_, pr-*ΔndhF* (◯) only showed slightly lower efficiencies when compared with all the assays with tobacco plants containing full functional *ndhF* gene. Conversely, the entropy produced (S_g_/E_BI_, lower boxes of **Figure 4**) decreased as the concentration of CO_2_ increased. The decrease above 400 ppm CO_2_ was less pronounced, especially for determinations in the intermediate 12 min (lower right box), for *ΔndhF* (□) and T181A (Δ; encircled; around 90% of the 360 ppm value) than for the other tobacco plants (around 75% of the 360 ppm value). Variable *ndhF* gene dose of pr-*ΔndhF* phenotypes in the different assays (◯) could explain the variable, although generally lower, efficiency at CO_2_ higher than 400 ppm of pr-*ΔndhF* than *ndhF* FC (▄) which, by containing the spectinomycin resistance gene, is more representative control than *wt* (●).

## DISCUSSION

As shown in **Figure 2**, the delay of Ndh complex-deficient *ΔndhF* tobacco plants in reaching high photosynthetic rates when exposed to sudden increases of light intensity becomes clear at CO_2_ concentrations above the 400 ppm predictable in the next decades. Under the sun fleck conditions common in open environments, the delay could impair the net photosynthetic performance of plants lacking *ndh* genes when compared with normal plants.

With the exception of the plastid *ndhF* gene disruption, no genome alteration has been found in *ΔndhF* tobacco (Martín et al., 2004, 2009; Zapata et al., 2005), which strongly indicates that the low proportion of *ndhF* gene copies and the resulting low level of the Ndh complex are solely responsible for the low photosynthetic performance of *ΔndhF* tobacco plants at high CO_2_ concentrations. The rapid photosynthetic response (Figure S3) of the plastid transgenic control *ndhF FC*, which has the same *aadA* gene insertion as *ΔndhF* but outside the *ndhF* reading frame, indicates that the *ΔndhF* phenotype is not due to an indirect effect of plastid transformation. The intermediate (although variable) photosynthetic performance of pr-*ΔndhF* tobacco plants (**Figures 2 and 4**) provides additional evidence of the necessity of the *ndh* genes and the thylakoid Ndh complex for rapid photosynthetic responses to light at high CO_2_ concentrations. On the other hand, the differences in the transpiration response (**Figure 3**) with regard to the photosynthetic response (**Figure 2**) to sudden light increases between *wt* and *ΔndhF* plants indicate that the *ndh* deficiency directly affects photosynthesis and is not mediated by a primary effect on the stoma machinery.

As described in preliminary results (Martín et al., 2009), the similar photosynthetic responses of *ΔndhF* and T181A at high CO_2_ concentrations suggests that the impossibility of site-181 phosporylation impairs the photosynthetic performance in T181A under rapidly fluctuating light intensities.

Redox balance of the electron transfer chain is critical for optimizing photosynthesis under fluctuating light in cyanobacteria and higher plants (Tikkanen et al., 2012; Allahverdiyeva et al., 2013). In *Chlamydomonas* (Alric, 2014), as in higher plants (Heber and Walker, 1992), redox equilibration is required for the maximal rate of cyclic electron flow. Therefore, the effect of the Ndh complex accelerating the photosynthetic response when light intensity suddenly increases could be due to its proposed role in optimizing the cyclic photosynthetic electron transport by adjusting the redox level of transporters (Casano et al., 2000; Joët et al., 2002). Electrons supplied by the Ndh complex prevent the over-oxidation of cyclic electron transporters that would occur due to the low supply of electrons from photosystem II between two high light intensity phases (**Figure 2**), especially at high CO_2_ concentrations that would rapidly deplete electrons from transporters by consuming NADPH in the Benson–Calvin cycle. In the case of *ΔndhF*, the supply of electrons from NADH through the Ndh complex fails, consequently the photosynthetic electron transporters remain over-oxidized and the rate of cyclic electron transport around photosystem I is low, impairing the thylakoid membrane potential and the ATP level required to rapidly reach high CO_2_ fixation rates in the following high light phase. As reported by Kanazawa and Kramer (2002), in comparison with the linear electron transport, the contribution of cyclic electron transport in maintaining thylakoid polarization is negligible at low CO_2_ concentrations, which could explain the similar photosynthetic rate responses between *ndh*-defective and *wt* tobacco plants at low CO_2_ concentrations.

The over-oxidation of cyclic electron transporters should be more pronounced the lower the light intensity and, accordingly, the delay in reaching a full photosynthetic rate at high CO_2_ concentrations in *ΔndhF* was longer for the 61 to 870 than for the 130 to 870 μmol m^-2^ s^-1^ PAR transition (**Figure 2**). Thus, the strong influence of the light intensity in the middle low intensity phase seems more compatible with a role of the Ndh complex improving cyclic electron transport by adjusting the redox level of transporters than by providing an additional cyclic electron transport chain, as proposed by Yamamoto et al. (2011), where the light intensity applied to the middle phase conceivably does not affect the subsequent photosynthetic rise of *ΔndhF*. The necessity to maintain the redox balance of the photosynthetic electron transport chain under fluctuating light has also been reported (Allahverdiyeva et al., 2013) in cyanobacteria, in which the flavo-diiron proteins Flv1 and Flv3 involved are dispensable under constant but not under fluctuating light conditions. Therefore, under fluctuating light, the inability of *ndh*-defective plants to provide enough electrons to balance the redox level of transporters (specially at high concentrations of CO_2_) could determine a low rate of cyclic electron transport under low light intensity. The low rate impairs the thylakoid polarization and ATP level required for a rapid response of the net photosynthetic rate when the light intensity suddenly increases in the following stage. Transitory shortage of ATP would decrease the rate of NADPH oxidation in the Benson–Calvin cycle at the next exposure to high light intensity, resulting in hyper-reduction of the electron transfer chain and PSI photodamage. Significantly, the PGR5 protein is also involved in the redox poising of photosynthetic electron transport (Nandha et al., 2007) and probably plays a role in the protection of PSI from photodamage under fluctuating light (Suorsa et al., 2013).

The effect of *ndh* gene products accelerating the increase of photosynthetic rates during the high intensity phases of fluctuating light reasonably indicates a definitive role for the Ndh complex. However, the mechanism involved and its relation with other factors, such as the PGR5 protein, require further investigations.

The differences in η and S_g_/E_BI_ between *wt* and *ΔndhF* were higher for the intermediate 12 min incubation than for the total 24 min incubation (**Figure 4**) and, plausibly, would be greater still under the rapid and more frequent changes of light in natural environments.

As the result of many assays, a 0.08 K^-1^ S_g_/E_BI_ value was estimated at 360 ppm CO_2_ and approximately 80 J K^-1^ was estimated for the S_g_ per leaf Kg and min, an amount 100-fold higher than the decrease of entropy associated to solute compartmentalization in cell organelles (Marín et al., 2009). Although roughly estimated, these values indicate that the *ndh*-associated S_g_ reduction in less than 1 s at CO_2_ concentrations higher than 400 ppm is in the range of structural leaf entropy values and could be evolutionarily significant as a selectable trait (Sabater, 2006) as found in other systems (Ding et al., 2011; Davies et al., 2013). In this regard, higher yields of energy conversions are equivalent to lower S_g_ and both are plausible selectable traits in photosynthesis.

## CONCLUDING REMARKS

The functional relevance of the thylakoid Ndh complex has been investigated by determining the photosynthetic response under fluctuating light and several CO_2_ concentrations in different tobacco plants affected in the plastid *ndhF* gene that encodes the NDH-F subunit of the Ndh complex. In contrast to *wt*, *ndh-* defective plants show an up to 5 min delay in reaching the maximum photosynthetic rate at CO_2_ concentrations higher than the ambient 360 ppm. Accordingly, *ndhF*-defective tobacco plants show a lower photosynthetic efficiency and higher S_g_ under rapidly fluctuating light intensities and high CO_2_ concentrations than *wt*. Based on our results and the previous results of other groups, it is postulated that the activity of the Ndh complex maintains high rates of photosynthetic cyclic electron transport by providing electrons to balance the redox level of transporters during the low light intensity stage and could be dispensable at low but not at high atmospheric concentrations of CO_2_ and under less intensively fluctuating lights. For the first time, these results establish a definitive connection between *ndh* gene products and photosynthetic performance and predict the influence of changing atmospheric CO_2_ concentrations on the evolutionary conservation of the *ndh* genes.

## AUTHOR CONTRIBUTIONS

Bartolomé Sabater, Mercedes Martín, and Dolores M. Noarbe conceived and designed research. Bartolomé Sabater, Mercedes Martín, and Patricia H. Serro conducted experiments. Bartolomé Sabater and Dolores M. Noarbe determined thermodynamics parameters. Bartolomé Sabater wrote the manuscript. Patricia H. Serro English edited the manuscript. All authors read and approved the manuscript.

## Conflict of Interest Statement

The authors declare that the research was conducted in the absence of any commercial or financial relationships that could be construed as a potential conflict of interest.

## Abbreviations

E′_BI_: Gibbs energy stored as biomass per mol CO2 fixed
PAR: photosynthetic active radiation
PCR: polymerase chain reaction
ppm: parts per million
S_g_: entropy production
η: photosynthetic efficiency
