# Supplementary material for: The rise of the photosynthetic rate when light intensity increases is delayed in ndh gene-defective tobacco at high but not at low CO2 concentrations
Source: Front Plant Sci. 2015 Feb 9;6:34. doi: 10.3389/fpls.2015.00034 (PMC4321573; doi:10.3389/fpls.2015.00034)
Supplement: Supplementary file 1 [file Data_Sheet_1.PDF]

**Supplementary Material** for ms.:

**The rise of the photosynthetic rate when light intensity increases is delayed in *ndh* gene-defective tobacco at high but not at low $CO_2$ concentrations**

Mercedes Martín, Dolores Marín, Patricia H. Serrot, Bartolomé Sabater (corresponding author, e-mail: bartolome.sabater@uah.es)

## Supplementary Materials and Methods

### *Transformation and selection of tobacco plastid transgenics*

*ΔndhF transgenic*

In Martín et al. (2004) we described how *ΔndhF* tobacco was obtained. A 1701 bp EcoRI restriction fragment of the tobacco plastid *ndhF* gene [starting from 17 nucleotides (nt) upstream to the initiation codon] was cloned into pBluescript SKII– vector (Stratagene, La Jolla, CA). A chimeric spectinomycin resistance conferring *aadA* gene, controlled by the tobacco *rrn*16 promoter and the *Chlamydomonas rbcL* 30 regulatory element (Koop et al., 1996), was inserted in the Eco47III site (nucleotide position 1023/1024 of *ndhF*) of the above-mentioned recombinant plasmid with opposite orientation to *ndhF*.

Leaves of 14-day-old sterile-grown tobacco seedlings were bombarded with plasmid DNA-coated gold particles using the PDS-1000 biolistic gun (Bio-Rad, Hercules, CA), and spectinomycin-resistant shoots were selected (Svab and Maliga, 1993). DNA isolated from primary regenerates was subjected to polymerase chain reaction (PCR) and Southern analysis to assess the correct integration of the transgene and homo-/heteroplastomy of spectinomycin resistant plants (Schmitz-Linneweber et al., 2001).

*T181A, T181D and ndhF FC (ndhF control, FC) transgenics*

In Martín et al., (2009) we described the preparation of these *ndhF* point mutants and control. The sequence interval between nucleotide positions 111,594 and 112,032 of the tobacco plastid genome (GenBank acession number Z00044) was amplified by PCR (Pfu, Fermentas) with primers F1S.439 and F1SalI from tobacco plastid DNA and cloned into pGEM-T Vector System I (Promega). The resulting recombinant plasmid (pGEM-F1) was linearized with SacII and a PCR-product (primers: aadA.SacII. for and aadA.rev) of the *aadA* expression cassette (Koop et al. 1996) was introduced, yielding the plasmid pGEM-F1-aadA. A second sequence interval of the tobacco plastid genome (position 112,033–114,762) was amplified by PCR with primers F2_5′_AatII and F2AatIIrev and cloned into the AatII site in the pGEM-F1-aadA plasmid. The resulting plasmid pGEMF1-aadA-F2 was sequenced for correct nucleotide sequence. For the codon substitutions in the *ndhF* gene changing the Thr-181 encoded in *wt* tobacco to Ala and Asp, the QuikChange Site-Directed Mutagenesis Kit (Stratagene) was used with the mutagenesis primers ndhF.T181A and ndhF.T181A.rev and ndhF.T181D.long and ndhF.T181D.longrev for T181A and T181D, respectively. No mutated plasmid pGEMF1-aadA-F2 was used for transformation to select the control *ndhF* FC transgenic tobacco maintaining the intact *ndhF* gene.

Leaves of 14-day-old sterile-grown tobacco seedlings were bombarded with plasmid DNA-coated gold particles using a biolistic gun (PDS-1000/He system, Bio-Rad, CA) and spectinomycin-resistant shoots were selected (Svab and Maliga, 1993). Plastid transformants were identified by PCR (aadA PCR (Primers: aadAre and aadAli59) and a PCR (AndhF and F2AatIIrev) to check the mutation by sequencing the PCR-product with the primer AndhF).

Primers:

F1S.439, CGGGGTACCGGTATTAGTCTGGATACGGC
F1SalI, ACGCGTCGACACAATTATAGCCTGTCTCTG
aadA.SacII.for, CGATCCGCGGAATTCGCCGTCGTTCAATG
aadA.rev, CCCGGGTACCGAGCTCCACC
F2_5′_AatII, TACGACGTCTAGAATTTGCTAATCGGCTG

F2AatIIrev, TCAGACGTCAATTGTGAATTAAAAAAAACAG
ndhF.T181A.rev, CTACACGATTAGCTACAAACGC
ndhF.T181A.for, GCGTTTGTAGCTAATCGTGTAG
ndhF.T181D.longfor, CTTGTCAAAAAGCGTTTGTAGATAATCGTGTAGGGGATTTTG
ndhF.T181D.longrev, CAAAATCCCCTACACGATTATCTACAAACGCTTTTTGACAAG
aadALI 59., TGCTGGCCGTACATTTGTACG
AndhF, TACGGTTGGAATTATGGTTC

Additional references

Schmitz-Linneweber, C., Tillich, M., Herrmann, R. G. and Maier, R. M. (2001). Heterologous, splicing-dependent RNA editing in chloroplasts: allotetraploidy provides trans-factors. *EMBO J.* 20, 4874–4883.

Svab, Z. and Maliga, P. (1993). High-frequency plastid transformation in tobacco by selection for a chimeric *aadA* gene, *Proc Natl Acad Sci USA*. 90, 913–917.

***Supplementary figures***

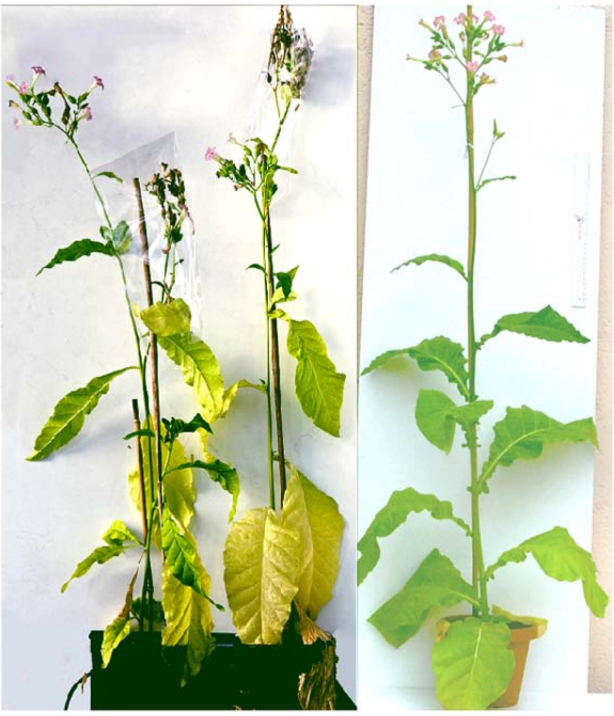


**Figure S1 Slight delay of leaf senescence in pr-*ΔndhF* tobacco in respect to *wt* tobacco**. In contrast to a clear delay of leaf senescence in *ΔndhF* tobacco, the photograph shows only a minor senescence delay in pr-*ΔndhF* when basal leaves of *wt* and pr-*ΔndhF* at similar stages of development are compared.

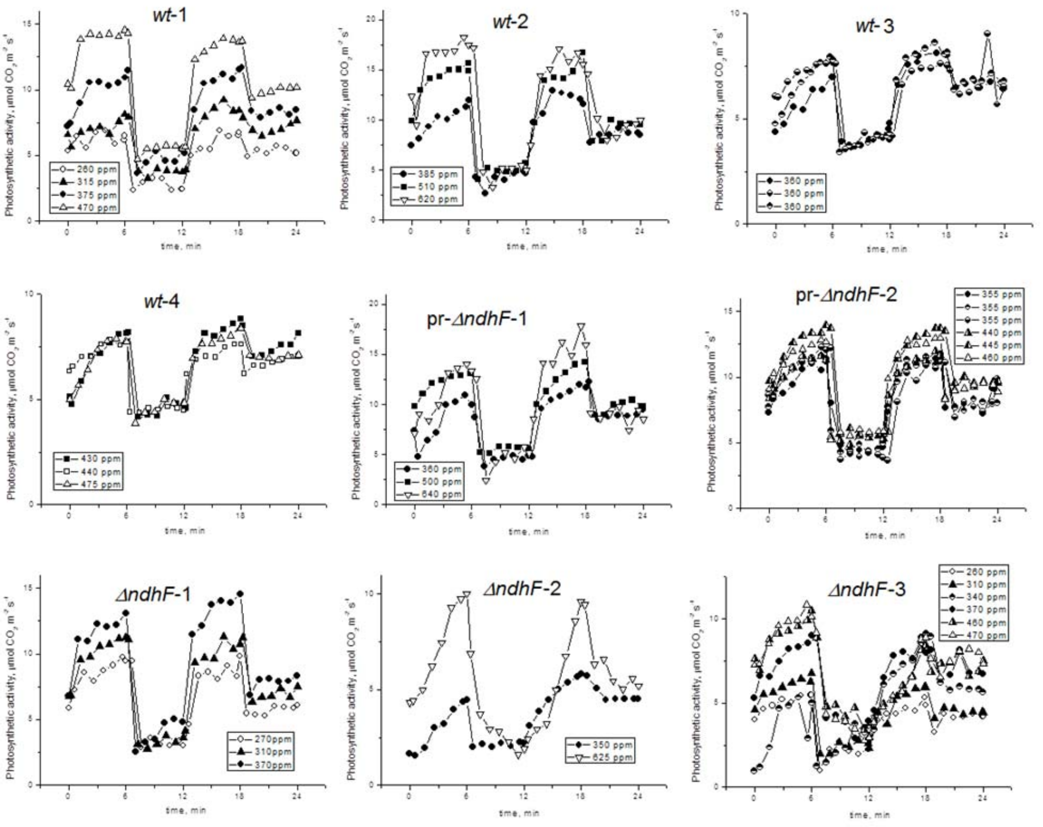


**Figure S2 Effect of the concentration of $CO_2$ on the photosynthetic rate under fluctuating light in different groups of assays with *wt, ΔndhF* and pr-*ΔndhF* partially reversed transgenic tobacco plants**. Figure shows graphics of different numbered experiments with tobacco tobacco. After a 15 min acclimation at 130 µmol $m^{-2}$ $s^{-1}$ PAR, the leaf region entrapped in the climatic chamber was subjected to successive six min periods (starting at 0 min time in the figure) of light intensities, abruptly changing according to the sequence 870, 61, 870 and 130 µmol $m^{-2}$ $s^{-1}$ PAR. The concentration of $CO_2$ varied for each sequence of light treatments as indicated.

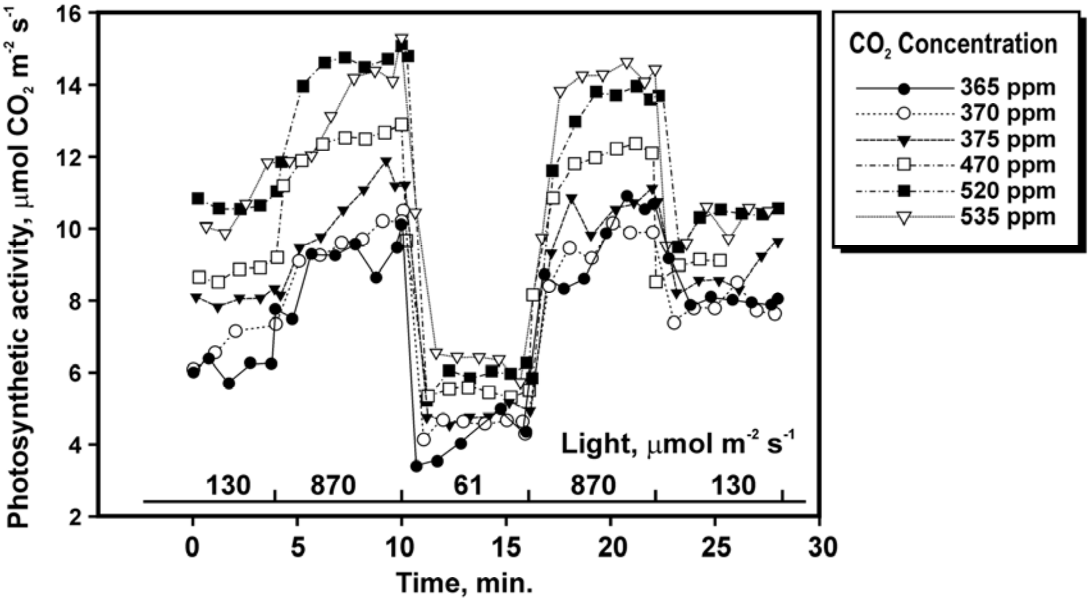


**Figure S3 Effect of the concentration of $CO_2$ on the photosynthetic rate under fluctuating light in the control transgenic *ndhF* FC tobacco**. Figure shows representative graphics of three different series of experiments. After a 15 min acclimation at 130 μmol $m^{-2}$ $s^{-1}$ PAR, the leaf region entrapped in the climatic chamber was subjected to successive six min periods (starting at 4 min time in the figure) of light intensities, abruptly changing according to the sequence 870, 61, 870 and 130 μmol $m^{-2}$ $s^{-1}$ PAR. The concentration of $CO_2$ varied for each sequence of light treatments as indicated.

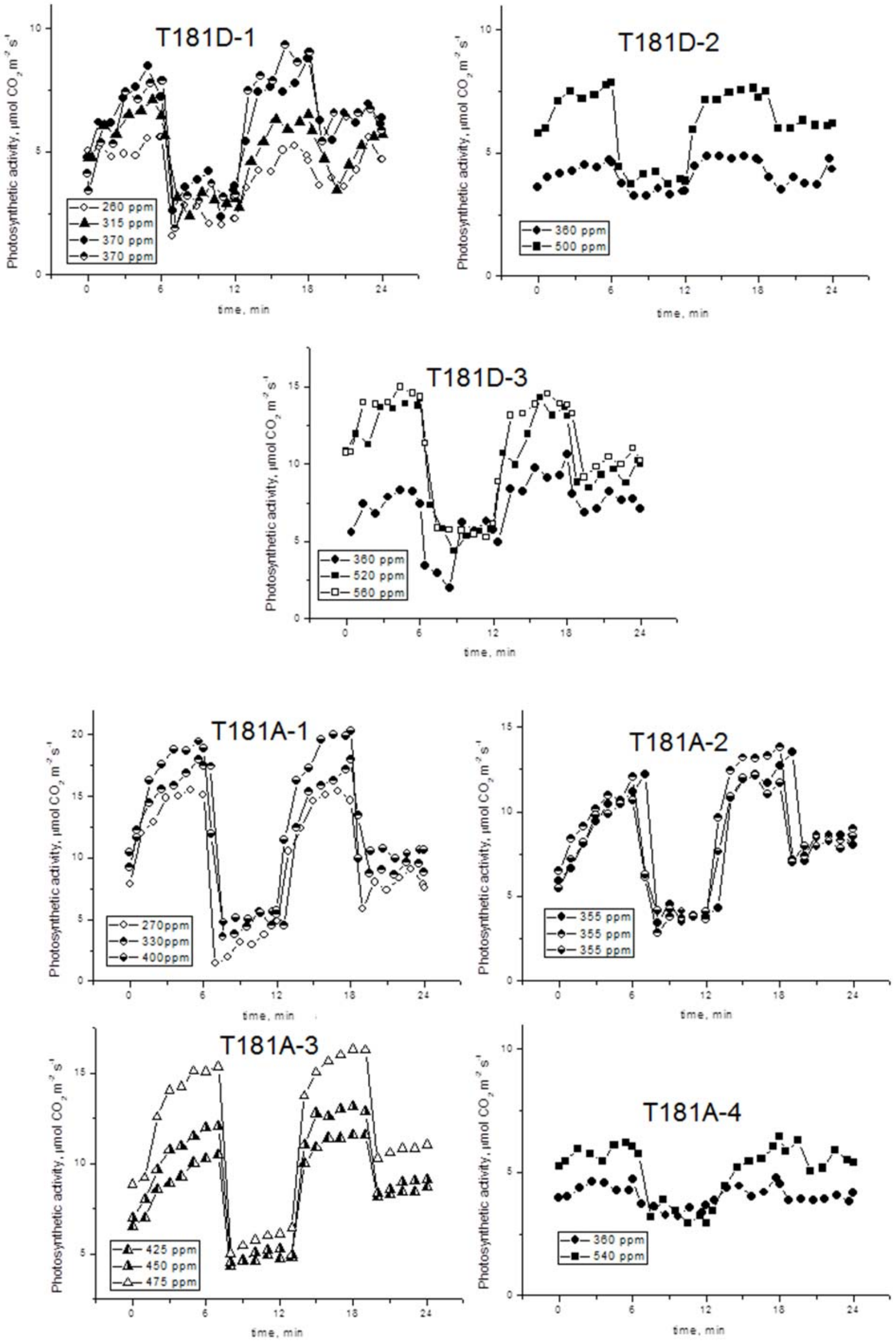


**Figure S4 Effect of the concentration of $CO_2$ on the photosynthetic rate under fluctuating light in different groups of assays with T181D and T181A transgenic tobacco plants**. Figure shows graphics of three numbered of experiments with T181D and four with T181A transgenic tobacco. After a 15 min acclimation at 130 µmol $m^{-2}$ $s^{-1}$ PAR, the leaf region entrapped in the climatic chamber was subjected to

successive six min periods (starting at 0 min time in the figure) of light intensities, abruptly changing according to the sequence 870, 61, 870 and 130 µmol $m^{-2}$ $s^{-1}$ PAR. The concentration of $CO_2$ varied for each sequence of light treatments as indicated.
